# Multivariate Classification of Major Depressive Disorder Using the Effective Connectivity and Functional Connectivity

**DOI:** 10.3389/fnins.2018.00038

**Published:** 2018-02-19

**Authors:** Xiangfei Geng, Junhai Xu, Baolin Liu, Yonggang Shi

**Affiliations:** ^1^Tianjin Key Laboratory of Cognitive Computing and Application, School of Computer Science and Technology, Tianjin University, Tianjin, China; ^2^Laboratory of Neural Imaging, Keck School of Medicine, USC Stevens Neuroimaging and Informatics Institute, University of Southern California, Los Angeles, CA, United States; ^3^State Key Laboratory of Intelligent Technology and Systems, National Laboratory for Information Science and Technology, Tsinghua University, Beijing, China

**Keywords:** major depressive disorder, pattern classification, functional connectivity, effective connectivity, spectral dynamic causal modeling

## Abstract

Major depressive disorder (MDD) is a mental disorder characterized by at least 2 weeks of low mood, which is present across most situations. Diagnosis of MDD using rest-state functional magnetic resonance imaging (fMRI) data faces many challenges due to the high dimensionality, small samples, noisy and individual variability. To our best knowledge, no studies aim at classification with effective connectivity and functional connectivity measures between MDD patients and healthy controls. In this study, we performed a data-driving classification analysis using the whole brain connectivity measures which included the functional connectivity from two brain templates and effective connectivity measures created by the default mode network (DMN), dorsal attention network (DAN), frontal-parietal network (FPN), and silence network (SN). Effective connectivity measures were extracted using spectral Dynamic Causal Modeling (spDCM) and transformed into a vectorial feature space. Linear Support Vector Machine (linear SVM), non-linear SVM, k-Nearest Neighbor (KNN), and Logistic Regression (LR) were used as the classifiers to identify the differences between MDD patients and healthy controls. Our results showed that the highest accuracy achieved 91.67% (*p* < 0.0001) when using 19 effective connections and 89.36% when using 6,650 functional connections. The functional connections with high discriminative power were mainly located within or across the whole brain resting-state networks while the discriminative effective connections located in several specific regions, such as posterior cingulate cortex (PCC), ventromedial prefrontal cortex (vmPFC), dorsal cingulate cortex (dACC), and inferior parietal lobes (IPL). To further compare the discriminative power of functional connections and effective connections, a classification analysis only using the functional connections from those four networks was conducted and the highest accuracy achieved 78.33% (*p* < 0.0001). Our study demonstrated that the effective connectivity measures might play a more important role than functional connectivity in exploring the alterations between patients and health controls and afford a better mechanistic interpretability. Moreover, our results showed a diagnostic potential of the effective connectivity for the diagnosis of MDD patients with high accuracies allowing for earlier prevention or intervention.

## Introduction

Major Depressive Disorder (MDD) is a mental disorder characterized by at least 2 weeks of low mood that is presented across most situations (Belmaker and Agam, 2008). The cause of MDD is complicated which includes psychological, environmental and genetic factors. The diagnosis of MDD is based on person's mental status examinations and experiences and the most widely used criteria are the Diagnostic and Statistical Manual of Mental Disorders (DSM-IV-TR) (American Psychiatric Association, 2000), World Health Organization's International Statistical Classification of Diseases and Related Health Problems (ICD-10) (World Health Organization, 1992). Those methods base on self-reported symptoms are easily impacted by human factors, which restricted the diagnosis and treatment of MDD in advance (Singh et al., 2010; Oyebode, 2013; Bordini et al., 2017). Undoubtedly, it is important to explore a more efficient and not based on self-reported symptoms method for MDD diagnosis.

In recent years, structure abnormalities in MDD have been reported at a group level in many studies (Kempton et al., 2011; Schmaal et al., 2017). Some specific brain regions in MDD patients, such as part of frontal regions, anterior cingulate, orbitofrontal cortex, hippocampus putamen, and caudate nucleus show different degree of volume reduction compared to HC (Lorenzetti et al., 2009; Zhao et al., 2017). The machine learning method especially multivariate pattern analysis (MVPA) has been used to explore structural and functional differences between MDD and healthy controls (HC), suggesting a promising direction on exploring an efficient diagnosis method for MDD based on neuroimaging data. MVPA is a widely used approach in task-related functional magnetic resonance imaging (fMRI) studies, which treats many consecutive voxels as a pattern and can improve the sensibility for imperceptible changes in brain activities (Weil and Rees, 2010). Based on those structure abnormalities between MDD patients and HC, many classification approaches based on MVPA are developed to examine whether those abnormalities can be treated as an objective biomarker for MDD diagnosis. With a support vector machine (SVM) and relevance vector machine (RVM) classifier, MDD patients were distinguished from HC with a 90% accuracy based on T1-weighted “structural” scans (Mwangi et al., 2012). A single SVM classifier was also used in a multi-ethnic structural neuroimaging data classification task and the best performance reached 78.3% (Sankar et al., 2016), which showed that the structure abnormalities cross the multi-ethnic group had a similarity pattern and machine learning methods can learn the pattern to afford classification abilities. The studies in structural neuroimaging data provide an important step for developing a potential efficient diagnosis method of MDD. However, there are some limitations when only structural neuroimaging data are used for MDD diagnosis. The main limitation is that there is a slow change in the structural abnormalities in MDD and only after a long period of time the structural abnormalities will become significant (Lorenzetti et al., 2009), which leads to lack of sensitivity for MDD diagnosis.

Except for structural neuroimaging data, functional neuroimaging data have attracted more and more attention in investigating the pathophysiology of MDD, as we can obtain the active brain status from the functional images. Our human brain is considered as an integrated network, which consists of anatomically separated but functionally linked brain regions (van den Heuvel and Hulshoff Pol, 2010). Many studies using the fMRI technique reveal that the pathophysiology of MDD involves a large-scale dysfunction in functional brain networks (Kaiser et al., 2015; Lv et al., 2016; Williams, 2017; Whitton et al., 2018). The functional connectivity (FC) is used as a most common measure for exploring the functional brain networks. FC, which represents the correlation between the time series of anatomically separated brain regions, has been used to investigate the dysfunctions in many mental disorder diseases, such as social anxiety disorder (Liao et al., 2010), depression disorder (Dørum et al., 2017). MDD patients showed decreased FC alterations in the bilateral amygdala, left anterior insula, left frontal pole, bilateral lingual gyrus comparing to HC (Veer et al., 2010). The resting-state networks distinctly at rest have been confirmed in many studies (Betzel et al., 2014; Nashiro et al., 2017), which mainly include the default mode network (DMN), visual network (VN), sensorimotor network (SMN), attention network (AN), salience network (SN) and fronto-parietal network (FPN) (van den Heuvel and Hulshoff Pol, 2010; Rosazza and Minati, 2011). DMN activates “by default” when a person is not involved in a specific task and deactivates during specific tasks, such as a cognitive task and attention task, which commonly consists of the medial prefrontal cortex (mPFC), bilateral inferior parietal lobule (LP) and posterior cingulate cortex (PCC) (Raichle, 2015). Besides, the altered dynamics functional brain networks in MDD patients have been reported in the dorsal attention network (DAN), FPN, and SN (Sambataro et al., 2017). SN consisting of the anterior cingulate cortex (ACC) and bilateral rostrolateral prefrontal cortex (RPFC), shows a significant increased power of the low frequency oscillations (LFO) during the task performance in MDD patients (Zhang et al., 2014). DAN, including the bilateral frontal eye field (FEF) and intraparietal sulcus (IPS), shows an altered amplitude of Low frequency oscillation (LFO) and reduced FC, revealing that the orienting attention dysfunction in DAN may be a possible pathophysiology of MDD (Corbetta et al., 2008). FPN, also referred to the executive network, is consisted of the bilateral prefrontal cortex (LPFC) and posterior parietal cortex (PPC) (Kaas et al., 2017; Ray et al., 2017). Ineffective transmission of information between prefrontal and parietal regions may be a main reason for MDD (Brzezicka, 2013). Many studies have found significantly altered connections in other resting-state network such as SMN and VN in MDD patients (Yao et al., 2009; Veer et al., 2010; Wei et al., 2015; Sambataro et al., 2017). Abnormal FC in MDD patients in the resting-state networks has been used as a biomarker for MDD diagnosis (Dørum et al., 2017). Using FC from 15 regions of interest (ROI) as features and SVM as a trained classifier for identifying MDD patients from HC, the accuracy achieved 83.3%, suggesting that resting-state FC can be used as a biomarker for MDD diagnosis (Craddock et al., 2009). Further more, different neurophysiological subtypes of depression were successfully distinguished only using FC in a MRI study (Drysdale et al., 2017), indicating the strong discriminative power of FC.

Although FC has a good performance in exploring the abnormalities of functional brain networks, it is not an efficient approach when we want to further understand the mechanism under the observed abnormalities, since FC simply represents the correlation between two brain regions. Effective connectivity (EC) is a more complex and efficient measure to examine the dynamic changes, which describes the causal influences that neural units exert over another (Friston, 1994). Using EC during an emotion-relevant task, the adolescents with MDD shows a significantly different connection from the amygdala to subgenual ACC (Schlösser et al., 2008). EC among several resting-state networks (such as DMN, AN, and FPN) altered significantly comparing to HC, which reveals that EC may be used as a biomarker for MDD diagnosis. Spectral Dynamic Causal Modeling (spDCM) is a model-based method to estimate the EC of the brain. It uses a plausible power-law model of the coupled dynamics of neuronal populations to generate the complex cross spectra among measured responses. It is similar to the conventional deterministic DCM for fMRI but models endogenous activities that would reproduce the functional connectivity observed in resting state fMRI (Friston et al., 2014).

FC has achieved good performances in exploring the abnormalities between patients and HC, which shows the potential as a biomarker in disease diagnosis in many studies (Craddock et al., 2009; Zeng et al., 2012). However, any hypothesis about coupling differences in brain cannot be valid throw FC. EC can reflect how the brain works by measuring the coupling among hidden brain states, it tries to explain observed dependencies, such as FC (Friston, 2011). It remain unknown whether EC can be used as an efficient and without self-reported symptoms biomarker for MDD diagnosis. In this study, we will explore the relationship and difference between FC and EC by using FC and EC as features to train a classifier for MDD diagnosis. Firstly, FC was extracted from a whole brain FC analysis, while spectral DCM was used to analysis the EC and the most important four testing-state networks (DMN, DAN, FPN and SN) for MDD were used to define the specific ROIs in this spectral DCM analysis. Linear SVM, non-linear SVM, k-nearest neighbors (KNN) and logistic regression (LR) classifiers were used to identify MDD patients from HC using the FC features and EC features separately. Based on the classification results and weight factor in classifiers, abnormal FC and EC in MDD patients will be discussed.

## Materials and methods

### Participants

Twenty four patients with MDD (16 females and 8 males, average age: 51.2 ± 10.6 years old, range 24–65 years old, PHQ is 21.1 ± 5.8, range 7–30, BDI is 32.3 ± 10.8, range 18–54, average time of education is 11.4 ± 3.4) and 24 HC (16 females and 8 males, average age: 47.8 ± 11.0 years old; range 25–65 years old, PHQ is 1.1 ± 1.2, range 0–5, BDI is 2.3 ± 1.4, range 0–5, average time of education is 13.1 ± 5.1) participated in this study. Those patients met criteria for DSM-IV-TR major depressive disorder without comorbidity and had a minimum duration of illness >3 months. All subjects were dominantly right-handed as determined by Edinburgh Handedness Inventory (Oldfield, 1971). Each participant provided written informed consent and the study was conducted in accordance with the local Ethics Committee.

### Data acquisition

Functional and structural images were obtained using 3T Siemens TIM Trio. Foam padding was used to minimize the head movement. One 8-min resting-state scan (240 time points, 36 axial slices, repetition time = 2,000 ms, echo time = 30 ms, voxel size = 3 × 3 × 3.99 mm^3^) was acquired on each participant for getting functional imaging data. A high-resolution T1-weighted scan (176 sagittal slices, voxel size = 1 × 1 × 1 mm^3^, repetition time = 20 ms, echo time = 6 ms) was acquired on each participant using T1-weighted sequence with generalized auto calibrating partially parallel acquisition.

### Data preprocessing

All fMRI data was preprocessed by the statistical parametric mapping software package (SPM12, http://www.fil.ion.ucl.ac.uk/spm/software/spm12). The first five volumes of scan data were discarded to allow the magnetization to approach dynamic equilibrium in each participant firstly. Each slice was corrected in slice timing by resampling slices to eliminate the time difference. Subsequently, a realignment analysis was performed with the middle image of the testing sequence as a reference; the data of each participant with a translation exceeding 3 mm and rotation exceeding 3 degree were removed. Individual structural images were linearly coregistered to the mean functional image, and then the transformed structural images were segmented into gray matter (GM), white matter (WM), and cerebrospinal fluid (CSF). Following this, all functional imaging data were normalized to Montreal Neurological Institutes (MNI) space and resampled to 3 × 3 × 3 mm^3^. Data were detrended and band-pass filtered (0.01 Hz < f < 0.08 Hz) and the sources of spurious variance, such as signals from WM, CSF and movement parameters, which extracted from the realignment process, were removed by a linear regression to remove artifacts and reduce physiological noise in CONN toolbox.

### Whole-brain functional connectivity analysis

A ROI-to-ROI functional connectivity analysis was performed using two whole-brain template separately, including the Automated Anatomical Labeling (AAL) template and Brainnetome template. AAL is a widely used anatomical template, which divides the whole brain into 78 cortical regions, 26 cerebellar regions and 12 subcortical regions according to anatomy (Tzouriomazoyer et al., 2002). The Brainnetome template contains more fine-grained functional brain subregions and gives more detailed anatomical information compared with AAL, because it is generated with both the functional connectivity and anatomical information (Fan et al., 2016). Brainnetome contains 246 subregions of bilateral hemispheres except for Cerebellum. Considering Cerebellum plays an import role in MDD (Lai and Wu, 2014; Guo et al., 2015), Cerebellum regions from the AAL template and Brainnetome were merge into a whole-brain template using the open source software WFU_pickatlas, which results a new template including 272 regions. In the following paper, we means this expanded template when we referred Brainnetome.

The following ROI-to-ROI functional analysis was performed using CONN toolbox (CONN17a, https://www.nitrc.org/projects/conn) in Matlab. The representative time series were first extracted by averaging the times series in each region and then the Pearson's correlation coefficients were calculated in each possible region pairs. A Fisher's r-to-z transform (Rosner, 2010) was applied to transform the correlation coefficients to the z-score space and then normalized to a standard normal distribution (0 mean, unit variance). This analysis generated a 116 × 116 matrix for the AAL template and a 272 × 272 matrix for the Brainnetome template, and the triangular portions of the two matrix were extracted separately and transformed to a vectorial feature space (6670 dimensions for the AAL template; 36,856 dimensions for the Brainnetome template).

### Effective connectivity analysis in resting-state networks

Four resting-state networks (DMN, DAN, FPN, and SN) were used in EC analysis. A seed-to-voxel FC analysis was performed using CONN toolbox to identify the main regions of the four resting-state networks. The seed ROIs, which were generated using a 6-mm radius spheres centered on MNI coordinates, were selected from previous studies (Vincent et al., 2008; Woodward et al., 2011). The seed ROIs were defined as follows: DMN (MPFC: 31 62 25); DAN (IPS_L:−18 29 43); FPN (LPFC: 17 54 35); SN (ACC: 31 50 37). For each seed, the temporal correlations between the seed and all other voxels in the brain were computed for each participant separately. A one-sample *t*-test statistical analysis was performed to define the coordinates of peak values in the four resting-state networks by masking with preexisting templates (Tsvetanov et al., 2016). Those coordinates were used in the following spDCM analysis.

The spDCM uses a plausible power-law model of the coupled dynamics of neuronal populations to generate the complex cross spectra among measured responses. It is similar to the conventional deterministic DCM for fMRI but models endogenous activities that would reproduce the functional connectivity observed in resting state fMRI (Friston et al., 2014). The spDCM analysis was performed using DCM12 in SPM12. First, volumes of interest (VOIs) for each network were defined as spheres with a radius of 6 mm centered at the MNI coordinates from the seed-to-voxel analysis, and then the first eigenvector was extracted after modeling the GLM that removed effects of the head motion and low-frequency drift. Then, the blood oxygen level-dependent fMRI time series was extracted from those VOIs. Subsequently, the extracted VOIs were used to construct DCM models. Four fully-connected models with bi-directional connections in each pair of VOIs were specified for DMN, DAN, FPN, and SN respectively. Then the optimal models for each resting-state network were obtained by using Bayes model selection (BMS) method. The post hoc function was used to perform the BMS, which adopted a greedy strategy to search over all permutations of the eight parameters whose removal produced the smallest reduction in model evidence when the free parameters equal or more than 16. There are 2^4^ = 16 free parameters for DMN, DAN, and FPN, respectively, and 9 free parameters for SN, which results 2^8^ = 256 DCM models for DMN, DAN, FPN, respectively and 2^9^ = 512 DCM models for SN. Then the model with the greatest model evidence was selected as the optimal model and the coupling parameters for the optimal model were estimated. Model parameters are extracted from four optimal models (each resting-state network has one) and transformed to a vectorial feature space. For an *n* node model, there are n^2^ free parameters. So we get 4^2^ × 3 + 3^2^ = 57 dimensions features for each participant, which were used for further classification analyzes.

### Feature selection

Given that there are some uninformative, irrelevant or redundant features, feature reductions can not only speed up the computation, but also improve the classification performance. Therefore, the feature selection step was utilized. First, One-sample *t*-test was applied to identify the significant connections among all participants and the features with the *p*-value bigger than 0.05 were removed. Subsequently, a univariate feature selection method (Gibbons and Kendall, 1990) was used to reduce the number of features. In this method, samples were divided into concordant and discordant pairs. Concordant pairs were defined as a pair of two-observation data sets (*x*_*mi*_, *y*_*m*_) and (*x*_*ni*_, *y*_*n*_), when they meet the following conditions:

(1) $$\begin{array}{r} signx\left(x_{mi}-x_{ni}\right)=signx\left(y_{n}-y_{m}\right) \end{array}$$

Where the signx is a signum function:

(2) $$signx\left(x\right)=\{\begin{array}{c} 1,\;if\;x>0\\ 0,\;if\;x=0\\ -1,\;if\;x<0 \end{array}$$

Correspondingly, it is a discordant pair when they meet the following conditions:

(3) $$\begin{array}{r} signx\left(x_{mi}-x_{ni}\right)=-signx\left(y_{n}-y_{m}\right) \end{array}$$

We defined the weight of i'th feature as the following formula:

(4) $$\begin{array}{r} w_{i}=\frac{\left|n_{c}-n_{d}\right|}{m\times n} \end{array}$$

Where there are m patients and *n* controls and *n*_*c*_, *n*_*d*_ represent the number of concordant and discordant pairs, respectively. Finally, we ranked the features according to *w*_*i*_ and selected the top k features as the final features set for the subsequent classification analysis. To obtain the optimizing features number (*k*-value) from numerous features, a rough searching analysis with a step 200 was performed in each classification process firstly to get the best performance interval range of *k*; then an accurate searching analysis of k with step 10 was performed in this interval range to identify the optimized feature number.

### Classification and evaluation

Four supervised learning classifiers (linear SVM, non-linear SVM, LR, and KNN) were used in the classification stage. This collocation is conducive to compare advantages and disadvantages of the different machine learning methods in fMRI data analysis (Misaki et al., 2010). The supervised learning classification process consists of two steps: training and testing. In the training step, the classifier finds a decision boundary that separates the samples in the input space using their class labels. Once the decision function is determined from the training set, it can be used to predict the class label of a new testing sample.

Training and testing classifiers on the same data could cause the overfitting problem (Dietterich, 1995). In this study, a leave-one-out cross-validation (LOOCV) procedure was used to avoid the over fitting and used as many as possible samples in the training step. In each LOOCV trial for *n* samples, n-1 samples were used for the training step and the leave-one sample was used for the testing step. The above feature selection process was wrapped in the LOOCV, because of the risk of overfitting. In each training step, the n-1 samples were first used to train the feature selection model and the training data and testing data were transformed to a new reduced feature space using the trained model. Then the training data after the feature selection analysis were used to train a classifier. In the testing step, the leave-one sample was transferred to the trained classifier and the predicted label of this sample was computed through the trained classifier. The accuracy, recall, specificity, f1 and receiver operating characteristic (ROC) curve were used to quantify the performance of a classifier. In this study, we referred the MDD patients as positive samples and HC as negative samples. TP represents the number of positive samples correctly classified; TN represents the number of negative samples correctly classified; FP represents the number of negative samples classified as positive samples; FN represents the number of positive samples classified as negative samples. The accuracy, recall, specificity and precision defined as:

(5) $$\begin{array}{r} accuracy=\frac{TP+TN}{TP+FP+FN+TN} \end{array}$$

(6) $$\begin{array}{r} recall=\;\frac{TP}{TP+FN} \end{array}$$

(7) $$\begin{array}{r} specifity=\frac{TN}{TN+FP} \end{array}$$

(8) $$\begin{array}{r} f1=\frac{2TP}{2TP+FP+FN} \end{array}$$

ROC, which is a curve created by plotting the true positive rate against false positive rate, can measure the diagnostic ability of a binary classifier. The area under the curve (AUC) is proportional to the performance of the classifier.

A permutation test was conducted to estimate the statistical significance of the observed classification accuracy. In the permutation test, all of the classification process were similar to the previous analysis except for the samples' label of the training set was randomly permuted. The statistical significance of a classifier was given as:

(9) $$\begin{array}{r} p=\;\frac{|acc\left(s^{\prime }\right)>acc\left(s\right)|+1}{m+1} \end{array}$$

Where m represents the permutation times, *acc*(*s*′)represents the accuracy on randomized permuted dataset and the *acc*(*s*) represents the accuracy obtained in normal classification process. In the current work, the m is set to 10,000. The bigger the *p*-value is, the more likely the accuracy is obtained by chance. The result was thought to be significant if *p* was <5% (*p* < 0.05). The whole classification and evaluation process was performed in python using scikit-learn toolkit, and detailed information about those conceptions can be found in the scikit-learn official website (http://scikit-learn.github.io/stable).

## Results

### Performances of the classification analysis

**Figure 1:**
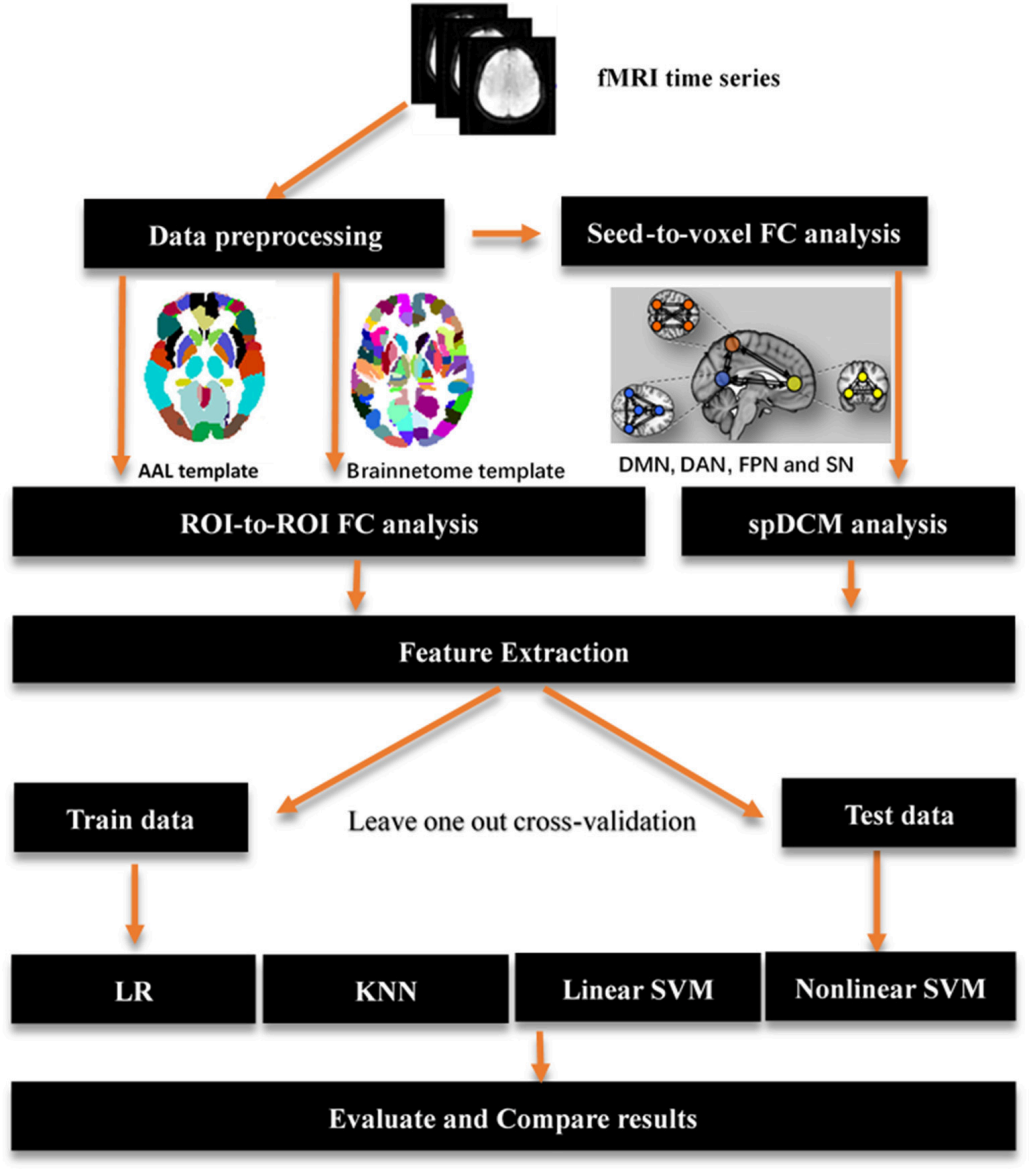
Overview of key processing steps for whole analysis. The origin fMRI time series first was preprocessing using SPM12, then a seed-to-voxel functional connectivity analysis performed on those data to identified main regions location of resting-state networks. Those regions were used to define spectral DCM models and get a best model fitted those data by using Bayes model selection method. AAL template and brainnetome template were used to perform a ROI-to-ROI functional connectivity analysis respectively. A feature extraction process was performed after functional connectivity analysis and spDCM analysis to prepare features for classification in the next step. Four classifiers were used in the classification with a leave one out cross-validation. AAL: Automated Anatomical Labeling; FC: functional connectivity; ROI: region of interest; SVM: support vector machine; LR: logical regression; KNN: k-nearest neighbors; spDCM: spectral Dynamic Causal Modeling; DMN: default mode network; DAN: dorsal attention network; FPN: fronto-parietal network; SN: salience network.

**Figure 2:**
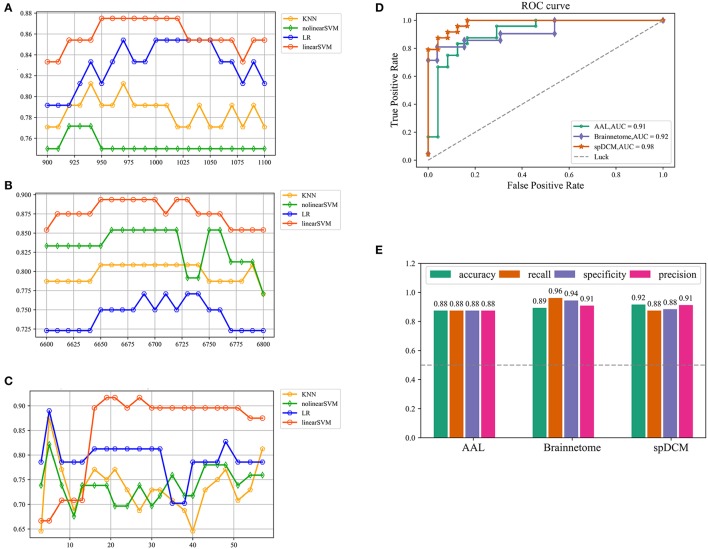
Classification results of three classification tasks. **(A,B,C)** Represents the research process of the hyper parameter k for AAL classification, Brainnetome classification and spDCM classification, respectively. For AAL classification and Brainnetome classification, the step of search is 10 while the step of search is 3 for spDCM classification considering the number of features and the speed of computation. **(D)** Represents ROC curve of the linear SVM classifiers in the three classification tasks. The gray dashed represents the chance level of a random classifier. **(E)** Represents the accuracy, recall, specificity, and f1 of linear SVM classifier in the three classification tasks. SVM: support vector machine; ROC: receiver operating characteristic.

In this study, three classification procedures based on the different templates were performed using four classifiers separately. Figure 1 showed the whole workflow of the three classification procedures. In the following paper, we referred the three classification process as AAL classification, Brainnetome classification and spDCM classification. The linear SVM classifier achieved the best performance among the four classifiers (linear SVM, nonlinear SVM, KNN, and LR) in all three classification tasks while the accuracies of the other classifiers were inconsistent. To further evaluate the performance of the linear SVM classifier in three classification procedures, the recall, specificity, f1, and ROC curve were calculated. The spDCM classification achieved the best performance (accuracy: 91.67%; f1: 91.30%; AUC: 0.98) when 19 effective connection features were used among the three classification tasks. The best performance for the AAL classification was observed when 950 functional connections were chosen as features (accuracy: 87.50%; f1: 87.50%; AUC: 0.91), and 6,650 functional connections as features were included for the Brainnetome classification (accuracy: 89.36%; f1: 90.90%; AUC: 0.92). The Brainnetome classification achieved the highest recall and f1 (recall: 96.15%; f1: 94.44%) among the three tasks, which revealed a good performance for the diseases diagnosis. Permutation tests were performed on all of the classifiers in the three classification procedures and *p*-values of all classifiers were <0.0001 (*p* < 0.0001), suggesting that accuracies of all of the classifiers were significantly higher than the chance level (50% of accuracy). The detailed results of the three classification procedures were showed in Figure 2.

### Spectral DCM analysis of the resting-state networks

The MNI coordinates of 15 specific regions for each group (MDD and HC) were obtained after the seed-to-voxel functional connectivity analysis. Based on those coordinates, time series of VOIs were extracted and used to define and estimate the DCM models. Details of those coordinates were listed in Table 1. DMN, DAN, FPN, and SN were used in this study and the first three resting-state networks consisted of four nodes (DMN: MPFC, PCC, LP_L, and LP_R; DAN: FEF_L, FEF_R, IPS_L, and IPS_R; FPN: LPFC_L, LPFC_R, PPC_L, and PPC_R) while SN included three nodes (ACC, RPFC_L, and RPFC_R). After the defined models were estimated, a Bayes model selection procedure was conducted to obtain the best model for each resting-state network. We found that the full connected DCM model achieved the best performance for each resting-state network, which was consistent with previous studies (Tsvetanov et al., 2016; Xu J. et al., 2017). The parameters of the full connected models were estimated and then transformed to a vector feature space, which resulted 4^2^ × 3 + 3^2^ = 57 dimensions of features for each participant. Those features were used in the following spDCM classification.

**Table 1:** Coordinates used in spDCM analysis.

**Resting-state network**	**Region**	**Center coordinates (HC group)**			**Center coordinates (MDD group)**		
		**x**	**y**	**z**	**x**	**y**	**z**
DMN	MPFC	3	57	−3	0	51	−6
DMN	PCC	6	−60	27	9	−51	30
DMN	LP_L	−48	−63	33	−45	−63	30
DMN	LP_R	51	−57	33	54	−63	30
DAN	FEF_L	−20	−6	64	−20	−6	64
DAN	FEF_R	20	−4	64	20	−10	64
DAN	IPS_L	−36	−44	50	−36	−46	48
DAN	IPS_R	38	−46	50	36	−46	48
FPN	LPFC_L	−42	36	30	−44	32	26
FPN	LPFC_R	44	38	28	44	32	26
FPN	PPC_L	−38	−48	52	−38	−50	52
FPN	PPC_R	38	−46	52	38	−52	52
SN	ACC	−2	20	36	0	20	32
SN	RPFC_L	−28	40	34	−32	42	30
SN	RPFC_R	30	38	34	30	44	30

*DMN: default mode network; DAN: dorsal attention network; FPN: fronto-parietal network; SN: salience network; MPFC: medial prefrontal cortex; PCC: posterior cingulate cortex; LP_L: left lateral parietal; LP_R: right lateral parietal; FEF_L: left frontal eye field; FEF_R: right frontal eye field; IPS_L: left intraparietal sulcus; IPS_R: right intraparietal sulcus; LPFC_L: left lateral prefrontal cortex; LPFC_R: right lateral prefrontal cortex; PPC_L: left posterior parietal cortex; PPC_R: right posterior parietal cortex; ACC: Anterior cingulate cortex; RPFC_L: left rostrolateral prefrontal cortex; RPFC_R: right rostrolateral prefrontal cortex*.

**Figure 3:**
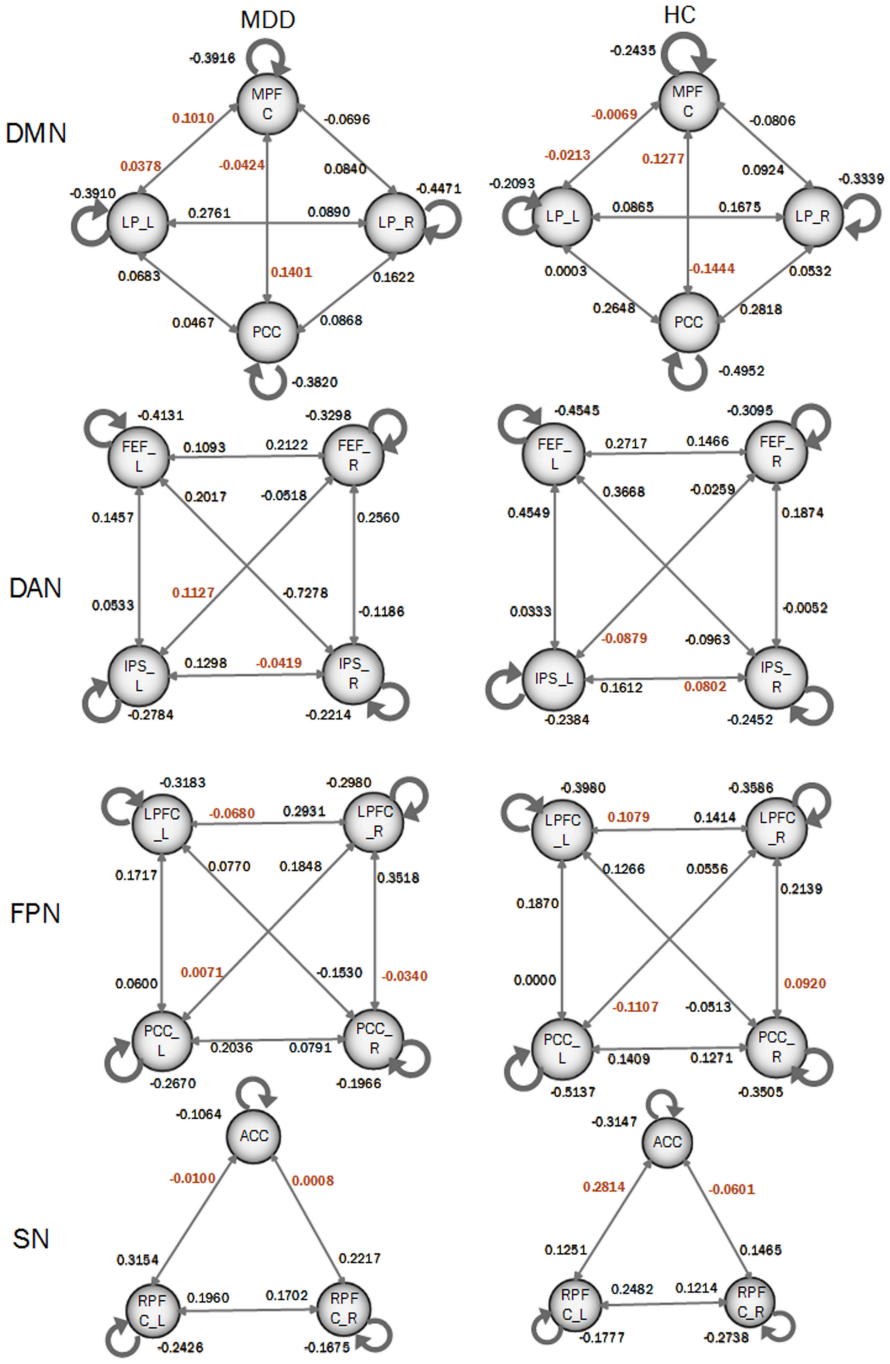
Coupling parameters of four DCM models obtained by bays model average. The connections colored by black paint not significant difference between groups. Connections colored by red paint between groups represents that they are significant difference between groups.

In order to compare the differences of DCM models between MDD patients and HC participants, a Bayes model average approach was adopted to obtain the parameters of four DCM models of four resting-state networks at a group level. Two-sample *t*-test was performed to identify the significance of the effective connectivity measures in two groups. As shown in Figure 3, there were significant differences in DCM model parameters, in which the intensities of some connections became positive in MDD while they were negative in HC participants. In details, those connections included: the bidirectional connections between MPFC and left LP, and both directions between MPFC and PCC; the connection from right FEF to left IPS and from left IPS to right IPS in DAN; the connections from right LPFC to left LPFC, from left PCC to right LPFC, and from right LPFC to right PCC in FPN; SN included the connections from left RPFC to ACC and that from right RPFC to ACC.

### High discriminative power of the functional connectivity measures

**Figure 4:**
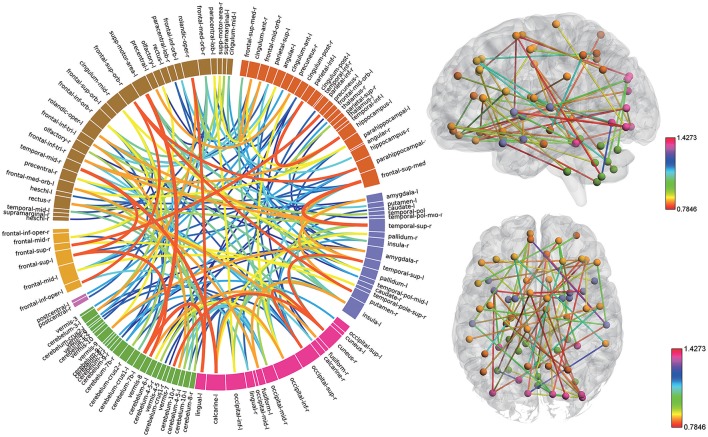
The ranking of regions weight and feature weight from classifiers of whole functional connectivity analysis using AAL template. In the left panel, the box in the ring represents brain regions and line connect two boxes represents functional connectivity of the two brain regions. Box size represents the size of the weight of brain regions. Color and z-depth of the line represents the size of the weight. In the right panel, small ball represents brain regions whose different colors means they belongs to different resting-state networks. Lines connect two small ball represents functional connectivity of two brain regions and its color represents the size of its weight.

**Figure 5:**
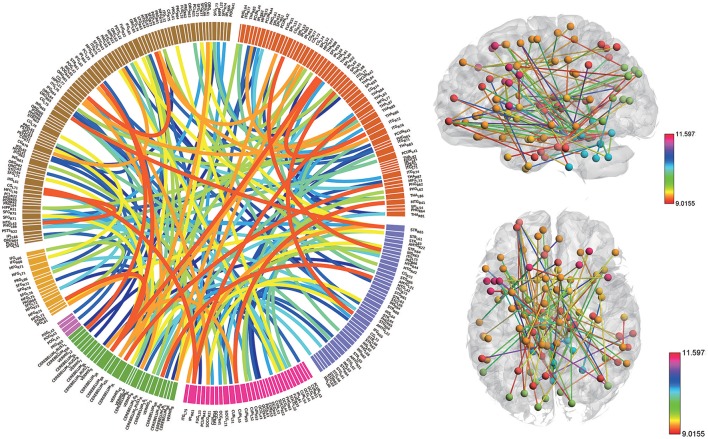
The ranking of regions weight and feature weight from classifiers of whole functional connectivity analysis using Brainnetome template. The meaning of color and legend is similar to Figure 4.

Consensus features, which were defined as the common selected features in each cross-validation fold, were identified and ranked according to their weights assigned by the linear SVM classifier, which generated 516 consensus features for the AAL classification and 5,596 consensus features for the Brainnetome classification. The region weight was defined as half of the weight of connections in those consensus features and was calculated separately for the AAL classification and Brainnetome classification. Those consensus features from both classification procedures were found to be mainly located in several brain regions, including the hippocampus, inferior parietal lobe, superior medial frontal gyrus, and precuneus in the left hemisphere, right parahippocampal, frontal middle orbital gyrus, superior parietal lobule, medial pre-frontal thalamus, posterior parietal thalamus, and inferior temporal gyrus in the right hemisphere, which belonged to the default model resting-state network; the bilateral superior temporal gyrus and amygdala, left insula, right ventral agranular insula, left angular, which belonged to the affective resting-state network; left calcarine, left lingual, right cuneus, right superior occipital gyrus, which belonged to the visual network, and the cerebellum regions such as left cerebellum 7b, right cerebellum 3, vermis 7, and vermis 8. For a better visualization of the consensus features and regions in the AAL classification and Brainnetome classification, a complex combination of graphs was created and shown in Figure 4 for AAL classification and in Figure 5 for Brainnetome classification. Colored lines in the two figures represent those consensus features while its color and thickness proportional to its weight assigned by the linear SVM classifier. Blocks in the edge of the circle represent the weight assigned by the linear SVM classifier.

Although the highest discriminative power connections in both classification procedures were mainly located in the several resting-state networks and cerebellum, there were slight differences due to the different parcellation of the two templates. The Brainnetome template is a more detailed division of the brain compared to the AAL template, which resulted more consensus features in Brainnetome classification. Besides, connections between the cerebellum and other regions in Brainnetome were assigned higher weights in the linear SVM classifier than AAL. The weights assigned by classifier were more evenly distributed in Brainnetome compared to that in AAL. We also found slight differences for the cross resting-state network connections in two classification procedures. The most cross networks connections in Brainnetome were the connections between the cerebellum and other networks while there was no significantly assemble connection in cerebellum for the cross resting-state network connections in AAL.

### High discriminative power of the effective connectivity measures

Fifteen consensus features in spDCM classification process were chosen, which could be divided into two groups: self-to-self connections and one-to-another connections. Those consensus features were ranked by their weights assigned by the linear SVM classifier. The left LP, left LPFC, left PPC, right RPFC, and right LRFC were assigned top 5 in the self-to-self connections. Those connections including the connections from left IPS to right IPS, from left LP to right PPC, from the left LPFC to right PPC, from left IPS to right left FEF, from left IPS to right FEF were assigned top 5 self-to-another connections. The details of this ranking were listed in Table 2. Region weight was defined as the half of the connections weight and the top 5 weighted regions included the left LPFC, left LP, right LPFC, left IPS, and left PPC. The detailed information of this ranking were listed in Table 3.

**Table 2:** Connections ranking of spDCM classification.

**Source region**	**Destination region**	**Weight**	**Resting-state network**
LP_L	LP_L	1.137816	DMN
LPFC_L	LPFC_L	1.056345	FPN
PPC_L	LPFC_L	0.929206	FPN
IPS_L	IPS_R	0.886355	DAN
LP_L	PCC	0.860112	DMN
LPFC_L	PPC_R	0.79765	FPN
IPS_L	FEF_L	0.758806	DAN
IPS_L	FEF_R	0.74761	DAN
RPFC_R	RPFC_R	0.624026	SN
LPFC_R	LPFC_R	0.582361	FPN
RPFC_L	ACC	0.569644	SN
PPC_L	PPC_L	0.469516	FPN
PPC_R	LPFC_R	0.457239	FPN
PCC	MPFC	0.420698	DMN
PPC_R	LPFC_L	0.415485	FPN

*DMN: default mode network; DAN: dorsal attention network; FPN: fronto-parietal network; SN: salience network; MPFC: medial prefrontal cortex; PCC: posterior cingulate cortex; LP_L: left lateral parietal; LP_R: right lateral parietal; FEF_L: left frontal eye field; FEF_R: right frontal eye field; IPS_L: left intraparietal sulcus; IPS_R: right intraparietal sulcus; LPFC_L: left lateral prefrontal cortex; LPFC_R: right lateral prefrontal cortex; PPC_L: left posterior parietal cortex; PPC_R: right posterior parietal cortex; ACC: Anterior cingulate cortex; RPFC_L: left rostrolateral prefrontal cortex; RPFC_R: right rostrolateral prefrontal cortex*.

**Table 3:** Ranking of regions weight of spDCM classification.

**Region**	**Weight**	**Resting-state network**
LPFC_L	2.332789	FPN
LP_L	1.567872	DMN
LPFC_R	1.21872	FPN
IPS_L	1.196385	DAN
PPC_L	1.136585	FPN
IPS_R	0.946298	DAN
PPC_R	0.835187	FPN
PCC	0.640405	DMN
RPFC_R	0.624026	SN
FEF_L	0.536289	DAN
FEF_R	0.373805	DAN
ACC	0.284822	SN
RPFC_L	0.284822	SN
MPFC	0.210349	DMN

*DMN: default mode network; DAN: dorsal attention network; FPN: fronto-parietal network; SN: salience network; MPFC: medial prefrontal cortex; PCC: posterior cingulate cortex; LP_L: left lateral parietal; LP_R: right lateral parietal; FEF_L: left frontal eye field; FEF_R: right frontal eye field; IPS_L: left intraparietal sulcus; IPS_R: right intraparietal sulcus; LPFC_L: left lateral prefrontal cortex; LPFC_R: right lateral prefrontal cortex; PPC_L: left posterior parietal cortex; PPC_R: right posterior parietal cortex; ACC: Anterior cingulate cortex; RPFC_L: left rostrolateral prefrontal cortex; RPFC_R: right rostrolateral prefrontal cortex*.

To further explore the advantages and disadvantages between FC and EC, a ROI-to-ROI functional connectivity analysis only using those ROIs used in spDCM classification was performed. This results 15 × 14/2 = 105 dimensions features. Following the same workflow in Figure 1, the functional connectivity features were extracted and send to classifiers and the classification results and each feature's weight in classifier was obtained. This classification was referred as resting-state network functional connectivity classification (RSNFCC) in the following paper. The best performance of RSNFCC achieved 78.33% accuracy (f1: 72.30%; AUC: 0.70) when using the top 55 features and linear SVM classifier. For further understanding of the relationship and difference between effective and functional connectivity, the connection parameter of FC and EC were plot in Figure S1 using the same form of organization.

## Discussion

In this study, both functional connectivity and effective connectivity were used as features to identify MDD patients from health populations using multivariate analysis methods. We found that effective connectivity achieved the best classification performance while functional connectivity with two templates slightly lower than effective connectivity. Both the functional connectivity and effective connectivity show a diagnostic potential for MDD disgnosis but the effective connectivity maybe more efficient comparing to functional connectivity.

### Altered functional connectivity in MDD

Altered functional connectivity in MDD was found in a majority of previous studies (Buchanan et al., 2014; Pannekoek et al., 2014; Kaiser et al., 2015; Dørum et al., 2017). Those altered FCs were mainly located in several resting-state networks and cerebellum. Bilateral hippocampus and parahippocampal gyrus, anterior cingulate cortex, thalamus, inferior temporal gyrus, posterior cingulate cortex, and medial prefrontal cortex, which belonged to default mode network, were considered to make great contributions in MDD (Gong and He, 2015). In this study, the left hippocampus, left inferior parietal lobe, right parahippocampal, medial pre-frontal thalamus, right parietal thalamus were assigned high weight by the linear SVM classifier, which were consistent with the previous study, suggesting that those regions played an important role in the pathophysiology of depression. The impaired FCs of DMN in MDD may be a main biomarker for MDD diagnosis. Disturbances within the fronto-parietal network are suggested to be strongly associated with the cognitive performance in patients with depression (Brzezicka, 2013). The abnormalities of FC in the fronto-parietal network have been reported in many MDD studies (Chen et al., 2017; Nord et al., 2017; Vasudev et al., 2017; Yu et al., 2017). There is one widely accepted hypothesis that the dorsal and lateral parts of the PFC are associated with more “cognitive” aspects of behavior, while the ventral and medial parts are mostly connected to “emotional” aspects of information processing (Brzezicka, 2013). One recent fMRI study found decreased activities in the parietal gyrus accompanied by diminished activities in the prefrontal cortex, and suggested the abnormalities within the dorsolateral prefrontal cortex (DLPFC)-parietal network during the working memory task. These studies revealed that the superior parietal cortex worked together with the prefrontal cortex and played a crucial role in cognitive neuroscience.

Besides the default model network, regions in other functional networks also shared higher weights in the classification procedure. For example, we found that the precuneus, parts of inferior temporal gyrus, middle frontal gyrus located in the attention network, parts of superior frontal gyrus involved in the FPN, and the amygdala, insula, middle temporal pole in the affective network, which were consistent with previous functional connectivity studies (Mayberg, 2003; Liu et al., 2010). The altered functional connectivity between the temporal pole and orbitofrontal cortex may reflect dysfunctions of visceral monitoring in depression (Liu et al., 2010; Sheline et al., 2010). Decreased functional connections between bilateral amygdala and left anterior insula were observed in this study, as suggested in a whole brain resting-state analysis (Veer et al., 2010). Dysfunctions in the limbic-cortical connectivity have been served as an important diagnostic marker for depression disorders (Mayberg, 2003). Using ReHo, MDD exhibited significantly decreased ReHo in the insula and in the cerebellum (Liu et al., 2010). In line with previous studies (Lai and Wu, 2016; Xu L.-Y. et al., 2017), we found that some cerebellum regions showed high discriminative powers, such as left cerebellum 7b, right cerebellum 3, vermis 7 and vermis 8, indicating the cerebellum as a key node in the cognitive processing of MDDs (Lu et al., 2012).

### Altered effective connectivity in resting-state networks in MDD

Functional connectivity can be used to describe the abnormal patterns of distributed activity, but it cannot tell us the influence that one neuronal system exerts over another (Harrison et al., 2003). Effective connectivity can describe the causal influences that neural units exert over another, which is more important for facilitating our understanding of ectopic foci leading to pathological conditions. Granger causality analysis (GCA), stochastic DCM and spDCM can be used to estimate the effective connectivity. GCA is a model-free, data-driven approach for effective connectivity analysis, which can model the effective connectivity of many nodes simultaneously. But application of GCA to resting state fMRI is particularly controversial (Craddock et al., 2013). Stochastic DCM can be used to analysis the effective connectivity in resting state fMRI, which is different from deterministic DCM because it do not ignore random fluctuations or noise on hidden states. The DCM can identify and quantify the effective connectivity that causes functional connectivity. The spDCM is designed for the resting-state fMRI effective connectivity analysis based on the observed functional connectivity, which is more accurate and more sensitive to the group difference compared to the stochastic DCM (Razi et al., 2015). The resting-state fMRI signals convey fluctuations in the low-frequency band typical within 0.01–0.08 Hz, and the low-frequency fluctuations are associated with the alterations of the externally-oriented and internally-oriented system (Wang et al., 2018). Furthermore, the effective connectivity estimated by the spDCM could reflect the time varying fluctuations in low-frequency neuronal states producing observed resting-state fMRI data by estimating the parameters of their cross correlation functions or cross spectra (Friston et al., 2014). In this study, spDCM was used to estimate the effective connectivity within four resting-state brain networks (Friston, 2011). Many studies have demonstrated that there was abnormal effective connectivity in MDD compared to HC group (Kasess et al., 2008; Vai et al., 2016; Li et al., 2017a; Wei et al., 2017).

DMN and FPN have been found to play an important role in the neuropathology of MDD. DMN has been linked to self-referential processing, while FPN has been linked to environmental information processing (Cieslik et al., 2011). In this study, effective connectivity from left LP to PCC and the self-to-self connection of left LP were assigned high weights, suggesting that they have the high discriminative power in distinguish MDD from health populations. The PCC is considered one of the hubs of the DMN with a general role in attention modulation, and in episodic and working memory. Declined influences from left LP to PCC in MDD compared with HC group have been reported in a recent study (Li et al., 2017b). This dysfunction of the connection may be related to the impaired signals propagate from one region to another in DMN. LP is associated with the episodic memory, and increased efferent connections from LP after treatment in MDD have been reported. By examining EC between amygdala and orbitomedial prefrontal cortex (OMPFC) during a happy and sad faces distinction task in both bipolar depressed (BD) and MDD patients, found that the bottom-up amygdala-OMPFC abnormalities of EC in the right hemisphere was found to be specific to bipolar disorder (Almeida et al., 2009), which revealed that the different pathophysiological mechanisms of the two type of depression. EC between four DMN nodes has been estimated by a spDCM method and used to explore the changes before and after 2-month treatment in MDD patients. MDD patients after treatment showed significant decreased effective connections from medial frontal cortex (MFC) toward PCC and toward right parietal cortex (RPC) and significant increased effective connections from the left parietal cortex (LPC) toward MFC, PCC, and RPC. This result reveals that MFC maybe play an important role in inhibitory conditioning of the DMN. In this study, we found that left LP shared a high discriminative power in distinguishing MDD, which may be related to the impaired memory function in MDD.

FPN, also referred as executive network, plays a pivotal role in control function, execution, and emotion processing. It seems to be strongly associated with cognitive problems in depression, especially those concerning executive functions. The dysfunctions within FPN are most probably connected to ineffective transmission of information between parietal and prefrontal regions (Brzezicka, 2013). Dysfunctions of the FPN in negative mood states of depression were found, which was consistent with our study. We found that the effective connections from left PPC to left LPFC, from left LPFC to right PPC, from right PPC to left LPFC showed high discriminative powers to identify MDDs from HCs. Bilateral LPFC were assigned high region weight by those connections. The left dorsal LPFC is well-known for top-down voluntary modulation of positive and negative emotions (Beauregard et al., 2001). Applying fast rTMS over the left PFC has been proved an antidepressant effect (George et al., 1997). The cortical circuit involving left frontal and right parietal regions is important in depression, and there were decreased functional connections between these regions in depression. The dysfunctions between the prefrontal and parietal regions may be a main reason that those effective connections were assigned higher weighs by the linear SVM classifier in the spDCM classification analysis.

There is evidence that MDDs showed increased attention for negative stimuli and decreased attention for positive stimuli (Disner et al., 2011). In this study, effective connections from left IPS to right IPS, left FEF, and right FEF were assigned high weights, revealing that those connections showed high discriminative powers in distinguishing MDD from health populations. IPS shows decreased brain activations in MDD compared to HC and is associated with the attention function. One recent study found that the left motor and the DAN showed reduced power in the low-frequency range in patients with MDD compared to healthy controls. Considering the role of DAN in orienting attention based on internal goals, those connections may contribute to the limited engagement with the external environment and potentially to increased self-reflection in MDD (Sambataro et al., 2017).

Our study found one effective connection from left RPFC to ACC in SN. Altered functional connectivity between ACC and frontal cortical regions in SN has been observed (Sheline et al., 2010). The role of dorsal ACC is mediating the integration of information across events in SN, so the effective connectivity from left RPFC to ACC may be associated with the dysfunction of information integration in MDD. SN is conceived as a toggle system allowing mental switching between processing self-referential and environmental information (Moran et al., 2013). Impaired salience responses to positive stimuli may be a main reason why MDD patients tend to make more concerns on the negative things. As dorsal ACC plays an important role in this switching, we speculate that the high discriminative connection from left RPFC to ACC in our study may be related to this dysfunction of switching (Yang et al., 2016).

### Different classification performances using functional connectivity and effective connectivity

Functional connections were applied as features in many classification studies, in which a high accuracy and good mechanistic interpretative power have been confirmed, but the classification performances using effective connections have not been explored. Effective connectivity can afford better mechanistic interpretative than functional connectivity, because it can model the causality interaction between two neurons. Using the effective connectivity as a biomarker, the pathophysiologic difference in the DMN was observed between bipolar depression and unipolar depression (Liu Y. et al., 2015), suggesting the potential ability of effective connectivity as a biomarker for MDD diagnosis. We suppose that effective connectivity will perform better than functional connectivity in the classification analysis.

To our best knowledge, this is the first study to compare the difference using EC and FC as features to identify patients from healthy controls. Effective connectivity and functional connectivity has closely relationship when using spDCM to estimate the effective connectivity. Functional connectivity adopts the cross-correlation function at zero lag as its measures while spDCM uses cross spectra, whose Fourier transforms correspond to cross-correlation function, to estimate the coupling parameters of DCM models. Cross spectra is a generalization of functional connectivity, so the effective connectivity estimated by spDCM preserve information on directed functional connectivity (Friston et al., 2014). In our study, spDCM classification indeed has a better performance compared with those classifications using functional connectivity as features. And the difference between MDD and HC was not significant for the functional connectivity, but there was significant difference in the effective connectivity. This reveals that effective connective may be more sensitive for detecting the group difference. Besides the more accuracy and more sensitive, the results obtained using effective connectivity can afford better mechanistic interpretative than functional connectivity, as the effective connectivity can provide the causality interaction of two neurons, while functional connectivity can only provide the correlation information of two brain regions.

### Differences of the classifiers performances in the classification analysis

In our study, we found that the linear SVM classifier achieved the best performance in all classification process, which suggested that the linear SVM was indeed a good classifier for fMRI data. This finding was consistent with majority of previous studies (Craddock et al., 2009; Wang et al., 2016; Saccà et al., 2017). For example, using the linear SVM classifier, patients with depression were successfully distinguished from healthy volunteers (R. Cameron Craddock), One recent study achieved high accuracy in object categories classification task using functional connections from task-related functional neuroimaging as features and SVM as the classifier. Besides, by comparing the classification performances of Random Forest and SVM in a prediction task of early multiple sclerosis, SVM was suggested to be better than Random Forest. Combining our findings with the previous studies, we speculated that the linear SVM classifier should be the preferred classifier for this type of fMRI data analysis.

### Limitations

One limitation is that we only explore inner-network effective connectivity and do not consider the causality interaction between networks. Dysfunction in the interaction between the FPN, DMN, and the DAN were reported in many previous studies (Buchanan et al., 2014; Sundermann et al., 2014; Wei et al., 2015). In the further work, we will add the cross-networks causal connection to feature space using spDCM to further investigate whether the accuracy would be improved and search a better combine of resting-state networks as biomarker for MDD diagnosis.

Another limitation is that the template used in whole-brain functional connectivity classification analysis has an important impact to the result. To explore the influence of the template in the whole-brain functional analysis, two templates (AAL and Brainnetome) were used in the whole-brain functional connectivity analysis. The classification performance of the Brainnetome template is slightly higher than AAL template, but more features (connections) were employed. We can see that through different templates to compute the FCs, there was no big change for the accuracy and the accuracy maintained at a higher value. The significant difference between the AAL and Brainnetome template was that weights assigned by the classifier were more evenly distributed in Brainnetome comparing to in AAL. As FCs were computed by averaging the times series of ROIs, the different division of brain regions have a great influence on the functional connections. One previous study explored the impact of different templates on the whole-brain functional connectivity analysis and developed a fine-gained atlas using spectral clustering method and achieved a good performance comparing with other templates (Craddock et al., 2012). It revealed that the choice of templates could impact the generated connections at a certain degree. Our study showed that the whole-brain functional connectivity classification maybe had a certain degree of stability although different templates were used, which need to be proved using more templates in future. Besides, the sample size in this study is not enough to totally prove that the effective connectivity is an efficient biomarker for MDD diagnosis, we use a leave-one-out cross-validation to use as many as sample in training and testing process and refer to the sample size of other papers (Zeng et al., 2012; Liu F. et al., 2015). We will use a larger sample size to verify our results in the future work.

## Conclusion

In this study, both functional connectivity and effective connectivity measures were used as features for the classification analysis. We found that both functional and effective connectivity show a diagnostic potential for MDD diagnosis, but effective connectivity may be more efficient compared with functional connectivity. The best performance achieved 91.67% accuracy when we used the effective connectivity estimated by spDCM using four resting-state networks. Those connections with high discriminative powers identified by the classifier can afford better mechanistic interpretative for the pathophysiology of MDD.

## Ethics statement

This study was carried out in accordance with the recommendations of Institutional Review Board (IRB) of Tianjin Key Laboratory of Cognitive Computing and Application, Tianjin University with written informed consent from all subjects. All subjects gave written informed consent in accordance with the Declaration of Helsinki. The protocol was approved by the Institutional Review Board (IRB) of Tianjin Key Laboratory of Cognitive Computing and Application, Tianjin University.

## Author contributions

BL designed the experiments. XG and JX performed the experiments. XG analyzed results. XG and JX wrote the manuscript. XG, JX, and YS contributed to manuscript revision. All authors contributed to discuss the results and have approved the final manuscript.

### Conflict of interest statement

The authors declare that the research was conducted in the absence of any commercial or financial relationships that could be construed as a potential conflict of interest.

## Supplementary material

Figure S1 The connection parameters of the functional and effective connectivity for MDD and HC groups. Fifteen regions of interest (ROI) defined in the DCM analysis were used for the functional connectivity analysis. The colorbar showed the connection intensity. MDD: major depressive disorder; HC: healthy control.
