# Supplementary figures and images for: Multivariate Classification of Major Depressive Disorder Using the Effective Connectivity and Functional Connectivity
Source: Front Neurosci. 2018 Feb 19;12:38. doi: 10.3389/fnins.2018.00038 (PMC5825897; doi:10.3389/fnins.2018.00038)

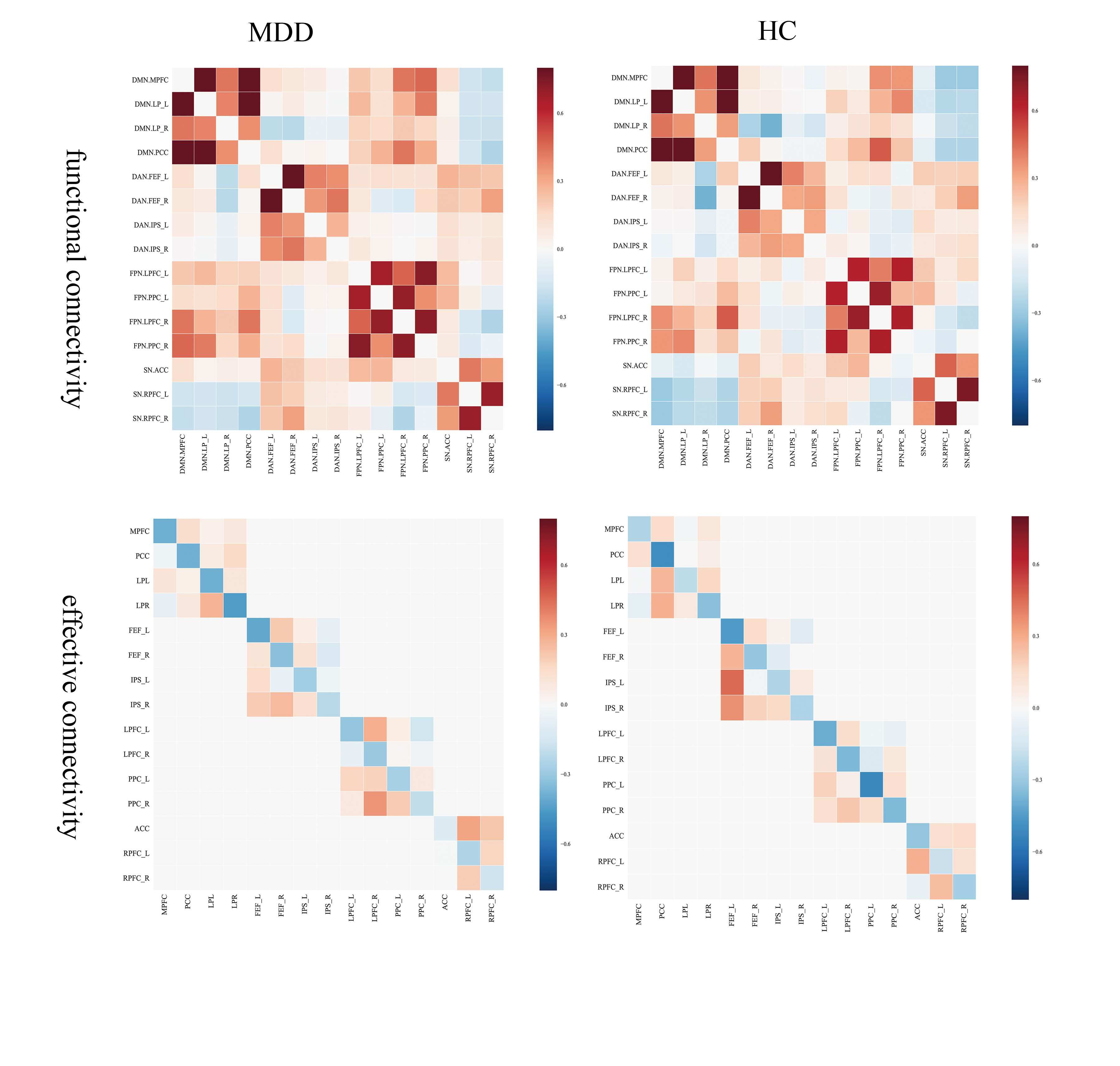

Supplement: Figure S1 — The connection parameters of the functional and effective connectivity for MDD and HC groups. Fifteen regions of interest (ROI) defined in the DCM analysis were used for the functional connectivity analysis. The colorbar showed the connection intensity. MDD: major depressive disorder; HC: healthy control. [file Image1.tif]
